# Ziemssen’s Cyclopœdia

**Published:** 1877-11

**Authors:** 


*Cyclopædia of the Practice of Medicine.* Edited by Dr. H. VON ZIEMSSEN, Professor of Clinical Medicine in Munich, Bavaria. Vol. XV. Diseases of the Kidney. By Prof. CARL BARTELS, of Kiel, and Prof. WILHELM EBSTEIN, of Goettingen. New York: William Wood & Co.

A work of nearly eight hundred pages is here presented the American profession upon diseases of the kidney alone. One reviewer characterizes this volume as the best of the series yet published. It contains more material that is new to most readers in our country than other volumes we have read, and it will doubtless be of service in helping towards clearness and accuracy of knowledge in a field in which considerable obscurity has prevailed. Our own writeis have not been quick to accept German and French pathology and pathological terms, though Niemeyer and several other foreign authors translated, and Loomis in his recent work, have presented the views in pathology which are current in Europe. We are glad to have this work on the kidneys, and hope it may be a means of progress.

Professor Bartels contributes the principal part of the work which is on the structural diseases of the kidneys and the general symptoms of renal affections. The passage of serum-albumen through the renal capillaries into the urine, he concludes, is due either to an abnormal increase of blood pressure, or to an altered state of the walls of the vessels, or a combination of these conditions. The albuminuria in all cases of uncomplicated cirrhosis of the kidney is solely due to increase of blood pressure in the malpighian bodies. Hypertrophy of the left ventricle of the heart which as a rule accompanies the process of contraction in the kidneys, lends additional force to the blood pressure, and while albumen is sometimes absent in localized renal effections, in the diffuse renal diseases it is chiefly the accompanying disturbance of circulation through the vessels of the kidneys, which, according to circumstances, produces transitory or persistent albuminuria. Urinary casts are enumerated as hyaline, epithelial or granular, waxy, or they are formed of coagulated blood, or are found in scarlet fever of ribbon shape or of peculiar form in the non-albuminous urine of icterus. As to the character of the homogeneous substance which enters into the formation of casts, he is not clearly convinced, and he does not indicate his opinion as to the extent to which epithelial secretion and fibrinous or albuminous transudation of the blood may effect or participate in their formation.

The subjects of albuminuria and of uremia are very interestingly and fully considered. He shows that urea or its derivative carbonate of ammonia, found in the blood is not alone satisfactory cause for the occurrence of uremia, but that uremic symptoms are nevertheless dependent upon disturbance of the functions of the kidney, and that thus uremia is a proper title for the disease.

The most important part of the work is that which treats of diffuse diseases of the kidney. Virchow's divisions are accepted by the author and his classification in full is;—1. Hyperemia, acute and passive; 2. Ischæmia, or the renal affection of cholera; 3. Parenchymatous inflammation, acute and chronic; 4. Interstitial inflammation or renal cirrhosis; 5. Amyloid degeneration. Under the head of hyperemia he considers the conditions produced in the kidney and urine after administration of cantharides or other substances which have irritative action upon that organ, and again the condition of the kidney in obstruction to the venous circulation from heart disease. The condition of the organ in cholera is thoroughly described.

In parenchymatous inflammation, the epithelial lining of the tubuli uriniferi is the part primarily affected. Acute parenchymatous inflammation of the kidney is the form which follows scarlet fever, diphtheria and other acute exanthemata in general. He says very little about the catarrhal, croupous and desquamative varieties of this disease, which have been described by other recent authors. It is rare for this acute affection to pass into a chronic one according to his observation. He has seldom observed in case of scarlatinal nephritis and has never known the nephritis of diphtheria to pass into chronic form; that which complicates rheumatism or follows taking cold is more likely to become chronic. Acute parenchymatous nephritis of pregnancy is treated separately.

Chronic parenchymatous nephritis is developed rarely from the acute form, but in the great majority of cases progresses in an insidious manner from the very beginning. He does not agree with others that this is a stage of parenchymatous nephritis that intervenes between the acute disease and a third or last stage of fibrous cicatricial contraction or atrophy. Chronic suppuration and marsh miasm are considered the most frequent causes of this disease: he has never known it to follow chronic alcoholism. Diaphoresis obtained by hot air treatment or Turkish bath he esteems of great value.

As to the causation of renal cirrhosis or interstitial nephritts, he finds considerable obscurity. In his experience chronic alcoholism has not been indicated as a cause, contrary to a wide-spread idea; possibly lead poisoning and gout may be causes, and he has never failed to find objective signs of hypertrophy of the left ventricle in his cases of this disease, and to confirm the same upon post mortem examination and holds that a diagnosis of the disease is not made certain until this complication is recognizable. He is entirely convinced of the individuality of this form of renal disease. Retinitis is most apt to complicate this form of diffuse nephritis.

Amyloid degeneration of the kidney was first described by Rokitansky in his Pathological Anatomy, published in 1842. It is invariably the local manifestation of grave constitutional disease, as long continued suppurative processes, scrofula, tuberculosis and syphilis. The spleen is almost always affected with amyloid change at the same time with the kidney and from its enlarged size and its firmness may be readily felt. The liver and the blood-vessels of the intestinal mucous membrane are also frequently affected with this same process.

The symptoms, course and treatment of parenchymatous, interstitial and amyloid renal disease are excellently presented, and the portion of the work embraced therewith, cannot fail to afford pleasure and benefit to its readers. Other affections of the kidney and ureters are treated of by Professor Ebstein in a satisfactory way, who has also considered the subject of amyloid degeneration and gives some facts additional to those before given. The chapter on nephrolithiasis is one of the most noticeable sections in this portion of the work.

